# A Retrospective Analysis of Short-Term Outcomes of Robotic and Laparoscopic Cholecystectomy: An Indian Tertiary Care Comparative Experience

**DOI:** 10.7759/cureus.69295

**Published:** 2024-09-12

**Authors:** Udipta Ray, Rahul Dhar

**Affiliations:** 1 Gastroenterology, Minimal Access and Bariatric Surgery, Fortis Hospital, Kolkata, IND; 2 Surgical Gastroenterology, Fortis Hospital, Kolkata, IND

**Keywords:** cholecystectomy, complications, laparoscopic, pain, robotic-assisted surgery

## Abstract

Background

There has been a gradual adoption of general surgery robotic programs in India. However, we still do not have a single comparative study reporting the initial experience of robotic cholecystectomy (RC) compared to laparoscopic cholecystectomy (LC). This retrospective study is aimed at addressing this clinical data gap.

Methods

This is a retrospective medical chart review where data related to patient demographics, and intraoperative and postoperative outcomes were collected. All patients underwent either RC or LC for gallstone disease, performed by a single surgeon from January 2020 to September 2023. The surgeon had passed the learning curve for RC and this data collection reflects his post-learning curve experience.

Results

A total of 100 cases (RC: 50; LC: 50) were collected. Baseline parameters such as age, sex, BMI, and comorbidities were comparable. There were no conversions from the planned procedure in either of the groups (0% vs 0%). There were no intraoperative complications such as bleeding or common bile duct injury (0% vs 0%). The rates of surgical site infections (SSIs) were numerically lower in the robotic group, 2% vs 6% (p = 0.3099). There were no postoperative complications in the robotic group, whereas one patient in the laparoscopic group experienced port side bleeding (0% vs 2%, p = 0.3173). The mean length of hospital stay was one day in both groups. The mean pain score 24- hours after the surgery was 1.78 ± 0.68 in the robotic group and 3.3 ± 1.2 in the laparoscopic group (p = <0.001). None of the patients required opioid analgesics in the robotic group, whereas 20% of patients in the laparoscopic group needed at least one dose of opioid analgesics (p = 0.0009). There were no reoperations reported in the robotic group, whereas the laparoscopic group reported 1 case. The 30-day mortality was nil in both groups.

Conclusion

RC is feasible in Indian settings. Compared to LC, it does not increase morbidity. The improvement in acute postoperative pain can potentially allow early ambulation and recovery. A larger multicentric study, comparing RC to LC in India will validate our initial experience.

## Introduction

Cholecystectomy is one of the most common general surgery procedures performed globally [1]. Since the 1990s, laparoscopic cholecystectomy (LC) has been the gold standard technique, offering significant improvement in morbidity, mortality, and hospital stay compared to the open approach [2]. From the early 2000s onwards, robotic surgery has also seen gradual adoption in both oncology and benign procedures. Robotic surgery provides enhanced ergonomics with wrist-like instrumentation, reduces tremors, and provides a stable 3D magnified visual field, potentially addressing some of the challenges encountered with laparoscopy [3]. It has been demonstrated in multiple studies that robotic cholecystectomy (RC) does not increase the morbidity of patients, compared to laparoscopy, when surgeons are in their learning curve (the amount of time or efforts it takes to learn a new procedure or number of procedures required to achieve the surgical proficiency) [1,4,5]. No difference, in terms of complications, has been reported in these studies. Beyond the learning curve of RC, recent systematic reviews and meta-analyses have reported comparable intraoperative and postoperative outcomes of RC and LC [1,6]. These studies report no significant difference in terms of intra- and postoperative complications, blood loss, surgical site infections (SSIs), or conversions. The operating room time in the RC group has been longer in these studies [1,6].

Studies have reported improved cosmesis satisfaction scores, body image perception scores, and pain scores after RC because of its precision, improved visualization, and faster recovery times [7,8]. From an Indian standpoint, RC is still in its infancy and its efficacy in these settings is largely unknown. To the best of our knowledge, this is the first study that compares RC to the gold standard, LC. As an extension of the above statement, we aim to report India’s first experience of RC and compare its 30-day perioperative outcomes to LC. The comparison is between multiport RC and multiport LC. This article was previously posted to the medRxiv preprint server on April 12, 2024.

A single skilled surgeon performed all of the procedures, LC and RC. The single surgeon has been performing LC for more than three decades. The surgeon has passed the learning curve for RC, and this data collection reflects his post-learning curve experience.

## Materials and methods

The study was conducted at a tertiary care institute in Kolkata, West Bengal, India. A retrospective medical chart review for the period of January 2020 to September 2023 was performed for consecutive patients who underwent noncomplex cholecystectomy using the laparoscopic or robotic approach for gallstone disease. Cholecystectomy cases for malignant disease or any other benign indications were excluded. Baseline characteristics such as age, sex, BMI, and comorbidities were recorded. Intraoperative parameters such as operating time, type of procedure (laparoscopic or robotic), and intraoperative complications were collected. Postoperative outcomes such as the length of hospital stay, analgesic usage, postoperative pain, the incidence of SSI or any other postoperative complications, rates of readmissions, and reoperations were recorded from medical records. Pain was measured by the 0 to 10 numerical rating scale (NRS) with a score of “0” indicating no pain and a score of “10” indicating the worst pain imaginable. The scale is one question and is easy to complete, easy to comprehend, reproducible, and can detect small changes in pain. The NRS was completed 24 hours post surgery. The study was conducted following the ethical principles outlined in the latest version of the Declaration of Helsinki, as well as the applicable good clinical practice guidelines, and ethical approval was obtained. The ethical approval for the study was obtained from the OrciVita Independent Ethics Committee vide letter number OIEC/10/02/2023 dated October 18, 2023. During the study, the institutional ethics committee of the institute was not registered with the regulatory authority; hence, an independent ethics committee approval was taken.

All operations (robotic or laparoscopic) were performed using the standard techniques advocated by surgeon societies, for multiport RC and multiport LC. Robotic surgery was performed using the Da Vinci Xi Surgical System (Intuitive Surgical, Sunnyvale, CA) using four ports. It consists of a 3D vision system and EndoWrist instruments with seven degrees of freedom to recreate dexterity and a range of movement for a high degree of precision and flexibility. A single skilled surgeon performed all the procedures, LC and RC. The single surgeon has been performing LC for more than three decades. The surgeon has passed the learning curve for RC (more than 20 cases over three months), and this data collection reflects his post-learning curve experience. The statistical analysis of the quantitative variables was summarized as the arithmetic mean with SD. Frequencies and percentages were used to summarize categorical data. A Pearson’s chi-square test or Fisher's exact test, as appropriate, was used to compare frequencies between the groups. The two-sample t-tests were used to compare differences in means between the robotic and laparoscopic groups. A two-sided p < 0.05 was considered statistically significant. Statistical analysis was performed using Stata 16.0 statistical software (StataCorp LLC, College Station, TX).

## Results

Perioperative outcomes

A total of 100 consecutive indicated noncomplex cholecystectomy cases were collected, 50 robotic cholecystectomy (RC) and 50 cases of LC. Age, BMI, and number of patients with symptomatic gallbladder were not significantly different between the two groups. The mean age in the RC group was 49.3 ± 17.37 years, whereas in the LC group, the mean age was 45.26 ± 8.73. Females represented 68% (n = 34) of cases in the LC arm compared to 32% of males in this group, whereas female representation was 62% (n = 31) in the RC group, with 38% being males. The mean BMI in the RC group was 26.83 ± 6.79, whereas the mean BMI in the LC group was 25.53 ± 3.35. All patients had symptomatic gallstone disease in both groups. None of the patients in either group had a previous history of upper abdominal surgery.

All 100 cases underwent noncomplex cholecystectomy for gallstone disease. The intraoperative and postoperative outcomes are summarized in Table 1.

**Table 1:** Perioperative outcomes *statistically significant; SD: standard deviation; statistical test: Pearson’s chi-square test or Fisher's exact test

Variable	Robotic-assisted (N=50)	Laparoscopic (N=50)	P value
Total procedure time, mean ± SD, min	20.7 ± 3.84	18.82 ± 2.135	0.0032*
Conversion to open cholecystectomy, n (%)	0 (0)	0 (0)	-
Intraoperative complications, n (%)	0 (0)	0 (0)	-
Surgical site infection, n (%)	1 (2)	3 (6)	0.3099
Postoperative complications, n (%)	0 (0)	1 (2)	0.3173
Clavien-Dindo classification of postoperative complications, n (%) Grade I	0 (0)	1 (2)	0.3173
Length of hospital stay, mean, days, mean	1	1	-
Pain score at 24 hours postsurgery, mean ± SD (measured by the 0 to 10 numerical rating scale, NRS)	1.78 ± 0.68	3.3 ± 1.2	<0.001*
Need for opioid analgesics, n (%)	0 (0)	10 (20)	0.0009*
Readmission within 30 days after surgery, n (%)	0 (0)	0 (0)	-
Reoperations, n (%)	0 (0)	1 (2)	0.3173
30-day mortality, n (%)	0 (0)	0 (0)	-

Total procedure time was significantly less in the LC group. However, this does not translate into any cost benefits as per the local healthcare practices. There were no conversions from the planned procedure to open cholecystectomy in either of the groups. There were no intraoperative complications such as bleeding or common bile duct injury. The rates of SSIs were numerically lower in the robotic group, 2% vs 6% (p = 0.3). There were no postoperative complications in the robotic group, where 1 patient in the laparoscopic group experienced port side bleeding. The mean length of hospital stay was one day in both groups.

The mean pain score at 24 hours after the surgery was 1.78 ± 0.68 in the robotic group and 3.3 ± 1.2 in the laparoscopic group. None of the patients required opioid analgesics in the robotic group whereas 20% of patients in the laparoscopic group needed at least one dose of opioid analgesics. There were no reoperations reported in the robotic group, whereas the laparoscopic group reported one case because of port-site bleeding. The 30-day mortality was nil in both groups.

## Discussion

RC has seen gradual adoption in Indian settings in the past two years. We undertook a retrospective medical chart review of 100 consecutive cholecystectomies, 50 patients each in the RC and LC group. We compared their first 30 days of perioperative clinical outcomes. For comparison, we do not have any comparative Indian studies and, hence, we are relying on global clinical studies to compare our Indian experience.

In terms of immediate perioperative clinical outcomes, we report comparable length of hospital stay and intraoperative and postoperative complications. There were no conversions in either group. Our findings are consistent with global literature. A 2023 systematic review of multiport LC and multiport RC included 14 studies and more than 3,000 patients [1]. This study reported no difference in blood loss intraoperatively (weighted mean difference was −6.73 mL, 95% CI: −16.31 to 2.84 mL for RC, with p = 0.17). The length of hospital stay was comparable, weighted mean difference of - 0.38, 95% CI: −0.87 to 0.12, and p = 0.13. There was no difference in overall postoperative complications, with a weighted odds ratio of 1.21 (95% CI: −0.80 to 1.84), with p = 0.86. Similarly, there was no significant difference in the incidence of bile duct injuries between the two groups, p = 0.46 [1].

We report significantly better pain scores 24 hours post surgery. The smaller incision and use of robotic arms to perform the surgery may have caused this. These findings are similar to those of Lee et al. [8]. In this study, patients who underwent RC reported lesser pain compared to LC, at six hours, 24 hours post surgery, two days post surgery, and one-week post-surgery time points [8]. However, this study compared single-port RC to multiport LC. A 2022 Korean study, comparing multiport RC to multiport LC reported significantly better pain scores in the RC group [9]. In this study, pain scores at two, four, and eight hours were significantly lower in the RC group (p = 0.04, 0.02, and 0.02, respectively) [9]. Moreover, the LC group received more analgesics after surgery (RC = 0.3 ± 0.5 vs. LC = 0.7 ± 0.9, p = 0.03). These are similar to our findings where none of the patients in the RC group needed opioid analgesics, and 20% of patients in the LC group needed at least one dose of opioid analgesics.

We observed an improving trend towards a lower incidence of SSI. Studies have not reported a significant difference in rates of SSIs [7,10,11]. Kudsi et al. [7] found the rates to be 2.4% in the RC group and 1.9% in the LC group (p = not significant), whereas Grochola et al. reported an SSI rate of 3.3% in both RC and LC groups [10].

None of the groups in our study reported a conversion of the planned procedure to open cholecystectomy. The systematic review by Straatman et al. reports a significantly lower risk of conversion in the robotic arm [1]. Multiple other studies have similarly reported a lower rate in the robotic arm [12,13]. A study by Tao et al. comparing multiport RC (n=171) to multiport LC (n = 441) showed a lower rate of conversion to open cholecystectomy in the RC arm, 0.6% vs 6.6% in the LC arm, p = 0.001 [12]. Similar to our study, the majority of cases in this study underwent noncomplex cholecystectomy. However, the mean age, BMI, and fraction of male cases were higher in this study compared to ours. One of the largest single-institution studies comparing RC to LC had conversion and risk of bile duct injury as the primary objectives [13]. In this analysis of 965 cases (RC, n = 676; LC, n = 289), the risk of conversion to open cholecystectomy was significantly lower in the RC arm (0.15%) compared to the LC arm (4.5%, p < 0.001) [13].

Prevention of bile duct injuries is key to a successful cholecystectomy. We did not observe any bile duct injury in our LC or RC cohort. However, a recent analysis of 10 years of Medicare administrative claims data by Kalata et al. has triggered a debate in the surgeon community [14]. They report a higher risk of bile duct injury, which would warrant operative repair within one year, in the RC arm (n = 25,084) compared to LC (n = 1,001,004). Multiple other studies have either reported a comparable incidence of bile duct injuries or a lower risk in the RC group [1,13,15,16].

We intend to report our data on one- and two-year rates of recurrence and reoperations in a follow-up study.

Strengths and limitations of our study

To the best of our knowledge, this is the first comparative study of RC and LC from India. This is also amongst a handful of global real-world studies comparing multiport RC to multiport LC. Only one expert surgeon performed and contributed cases to this study, and, thus, there was uniformity in the operative techniques. However, this could also be considered one of the limitations because of the potential for bias in the interpretation. The other limitations were the retrospective design without any formal sample size or power calculations and the lack of follow-up for reporting the medium to long-term results. Furthermore, we were also unable to do a cost-effective analysis because of data limitations.

## Conclusions

Our study presents the first comparative Indian experience for RC. In our study, the robotic approach has shown encouraging short-term perioperative outcomes, comparable to the gold standard LC. Future studies, with larger sample sizes and prospective designs, should compare the two approaches. Additionally, multicentric data on long-term clinical outcomes such as recurrences and reoperations need to be generated to evaluate the real-world effectiveness of robotic-assisted surgery in Indian settings.
